# Probing the Potential of Defense Response-Associated Genes for Predicting the Progression, Prognosis, and Immune Microenvironment of Osteosarcoma

**DOI:** 10.3390/cancers15082405

**Published:** 2023-04-21

**Authors:** Liangkun Huang, Fei Sun, Zilin Liu, Wenyi Jin, Yubiao Zhang, Junwen Chen, Changheng Zhong, Wanting Liang, Hao Peng

**Affiliations:** 1 Department of Orthopedics Surgery, Renmin Hospital of Wuhan University, Wuhan 430060, China; lkh1227@whu.edu.cn (L.H.);; 2 Department of Biomedical Sciences, College of Veterinary Medicine and Life Sciences, City University of Hong Kong, Kowloon Tong, Hong Kong SAR, China; 3 Department of Clinical Medicine, Xianyue Hospital of Xiamen Medical College, Xiamen 310058, China

**Keywords:** defense response, osteosarcoma, prognosis, metastasis, immune, therapy

## Abstract

**Simple Summary:**

Osteosarcoma (OS) is the most common primary orthopedic malignancy and typically affects children and young adults. Its lesions often metastasize to distant sites in the body, such as the lungs. Metastatic OS frequently recurs and has a poor prognosis. Our main objective of this study is to provide new insights into the clinical management of patients with osteosarcoma and to explore the risk factors affecting osteosarcoma metastasis. We established a biological marker consisting of three genes from the perspective of defense response for the first time to predict the prognosis of osteosarcoma and to discover new treatment methods. Our findings have implications for both the clinical management and future research of osteosarcoma.

**Abstract:**

Background: The defense response is a type of self-protective response of the body that protects it from damage by pathogenic factors. Although these reactions make important contributions to the occurrence and development of tumors, the role they play in osteosarcoma (OS), particularly in the immune microenvironment, remains unpredictable. Methods: This study included the clinical information and transcriptomic data of 84 osteosarcoma samples and the microarray data of 12 mesenchymal stem cell samples and 84 osteosarcoma samples. We obtained 129 differentially expressed genes related to the defense response (DRGs) by taking the intersection of differentially expressed genes with genes involved in the defense response pathway, and prognostic genes were screened using univariate Cox regression. Least absolute shrinkage and selection operator (LASSO) penalized Cox regression and multivariate Cox regression were then used to establish a DRG prognostic signature (DGPS) via the stepwise method. DGPS performance was examined using independent prognostic analysis, survival curves, and receiver operating characteristic (ROC) curves. In addition, the molecular and immune mechanisms of adverse prognosis in high-risk populations identified by DGPS were elucidated. The results were well verified by experiments. Result: BNIP3, PTGIS, and ZYX were identified as the most important DRGs for OS progression (hazard ratios of 2.044, 1.485, and 0.189, respectively). DGPS demonstrated outstanding performance in the prediction of OS prognosis (area under the curve (AUC) values of 0.842 and 0.787 in the training and test sets, respectively, adj-*p* < 0.05 in the survival curve). DGPS also performed better than a recent clinical prognostic approach with an AUC value of only 0.674 [metastasis], which was certified in the subsequent experimental results. These three genes regulate several key biological processes, including immune receptor activity and T cell activation, and they also reduce the infiltration of some immune cells, such as B cells, CD8+ T cells, and macrophages. Encouragingly, we found that DGPS was associated with sensitivity to chemotherapeutic drugs including JNK Inhibitor VIII, TGX221, MP470, and SB52334. Finally, we verified the effect of BNIP3 on apoptosis, proliferation, and migration of osteosarcoma cells through experiments. Conclusions: This study elucidated the role and mechanism of BNIP3, PTGIS, and ZYX in OS progression and was well verified by the experimental results, enabling reliable prognostic means and treatment strategies to be proposed for OS patients.

## 1. Introduction

The defense response is the innate function of the body for resisting invasion by internal and external pathogenic factors, which appear with the existence of a foreign body or injury, and helps limit damage to the body or facilitate the promotion of injury recovery. A variety of pathogenic factors and foreign bodies often threaten the human body, and the results caused by the body’s immune response may also result in damage to the body [1,2]. Immune cells are involved in constituting the tumor microenvironment (TME) from the early stage of tumor formation. Immune cells can recognize tumor-specific antigens and activate the immune system, and the coordination of natural and acquired immune cells can generate an efficient anti-tumor immune response, while tumor cells also have different immunosuppressive mechanisms to counteract the anti-tumor immune response [3]. At the same time, the body possesses a complete defense system that can protect it from potential damage by disease-causing agents. The body’s defense response can influence tumorigenesis and progression. Several studies have identified an association between different tumors and defense response-related genes [4,5,6,7]. Defense response occurrence helps the body to kill tumor cells and prevents their escape [8,9,10]. Therefore, there is a close relationship between the development of both defense response-related genes and tumors.

Osteosarcoma (OS) is a malignant tumor that generally occurs in children and young adults [6,7]. Osteosarcoma often metastasizes to various parts of the body, including the lungs. Metastatic OS often relapses and the prognosis is generally poor [8]. As neoadjuvant chemotherapy and other therapies have developed in recent decades, the five-year survival rate of osteosarcoma has reached 70% [11]. However, the overall five-year survival rate for patients who are diagnosed with early lung metastasis is below 20% [12], and prognostic factors and appropriate treatment for metastatic OS patients have yet to be determined [13,14]. Therefore, exploring a new prognostic method to further improve the prognosis of osteosarcoma patients is essential.

A correlation may exist between the defense response of the body and tumor occurrence and development. A tumor is a foreign body that stimulates the human body to produce a corresponding defense response. There are many studies exploring the relationship between different tumors and the defense response, but there are few relevant studies in osteosarcoma. The aim of this study was to explore the potential correlation between the defense response of the body and osteosarcoma occurrence and development. A completely new prognostic prediction method and treatment strategy for osteosarcoma was obtained, and relevant experimental verification was conducted as a means of providing new ideas for osteosarcoma treatment and scientific research.

## 2. Materials and Methods

### 2.1. Data Collection

We obtained 84 osteosarcoma samples with both clinical information and transcriptomic data from the TARGET database (https://xena.ucsc.edu/, accessed on 6 April 2023), and we obtained microarray data for 12 MSC samples and 84 osteosarcoma samples from the GSE33383 dataset (GPL10295 platform) from the GEO database (https://www.ncbi.nlm.nih.gov/geo/, accessed on 6 April 2023). The collected clinical information included survival status, survival time, metastasis, sex, and age. The tumor samples in the TARGET database were randomly assigned to the training (*n* = 50) and test (*n* = 34) sets using R software (version 4.1.2).

### 2.2. Acquisition of Defense Response-Associated Differential Genes

We selected 12 mesenchymal stem cell samples and 84 osteosarcoma cell samples from the GPL10295 platform in dataset GSE33383 for differentially expressed gene (DEG) screening, and mesenchymal stem cell samples were selected as the control group for osteosarcoma samples. The R package “limma” was then used to identify DEGs in GSE33383; the criterion was determined by fold-change (set as 1) and adjusted *p* value (set as 0.05). The differentially expressed genes were used for GSEA analysis and they were found to be significantly enriched in the Gene Ontology (GO) DEFENSE RESPONSE pathway. In order to obtain DRGs, the gene set of this pathway was intersected with the differentially expressed genes. The R package “pheatmap”(version 1.0.12) was used to draw the DRG expression heatmaps of normal samples and tumor tissue samples.

### 2.3. Construction of a Prognostic DRGs Signature

The R package “survival” (version 3.5–5) was used to perform univariate Cox regression for each DRG using the survival data. Then, LASSO regression was performed to avoid overfitting. The optimal and minimum criteria for the penalty (λ) were selected with 10 times cross-validation. Then, we used multivariate Cox regression analysis to identify the prognostic DRGs, and DGPS was constructed. These prognosis-related DRGs were utilized to construct an equation (DGPS) to calculate risk scores of osteosarcoma samples: $RiskScore=\sum_{i=1}^{n}Coef_{i}^{*}X_{i}$, where $Coef_{i}^{*}$ represents the coefficient (risk factor values for different genes) and Xi represents the normalized count of the DRG (gene expression). Risk scores were calculated for each osteosarcoma sample based only on the expression of different DRGs (i.e., the equation described above), and the median risk score of all osteosarcoma samples was used as the basis for classifying risk subgroups as high-risk (above the median score) and low-risk (below the median score).

### 2.4. Validation of DGPS

The Kaplan–Meier (KM) method was used to demonstrate the survival prediction value of DGPS. The accuracy and diagnostic value of DGPS were assessed using ROC curves and AUC. Principal component analysis (PCA) was performed to verify DGPS, and the R package “scatterplot3d” was used for visualization. The consistency index (C-index) was used for predicting the precision of DGPS with R packages “dplyr”, “survival”, “pec”, and “rms”. The test group and all cohorts were used in the validation of this model.

### 2.5. Exploration of the Relationship between DGPS and Clinical Features

To assess the suitability of DGPS for osteosarcoma with various clinical features, we used univariate and multivariate Cox regression analyses on 84 osteosarcoma samples from the TARGET database (https://xena.ucsc.edu/, accessed on 6 April 2023) to explore the association between DGPS and gender, age, and metastasis in order to reveal their potential role in OS.

### 2.6. Nomogram Construction of DGPS and Clinical Characteristics

Univariate and multivariate Cox regression were performed on 84 osteosarcoma samples from the TARGET database (https://xena.ucsc.edu/, accessed on 6 April 2023) in order to study the independent prognostic role of DGPS. A nomogram was then developed using the R packages “regplot” (version 1.1), “rms” (version 6.6-0), and “survival” (version 3.5–5). A calibration curve was then constructed to verify its precision.

### 2.7. Exploration of the Relationship between Model Genes and OS Metastasis

A box plot was used to explore the relationship between BNIP3 and OS metastasis, and then ROC, AUC, and a correlation scatter diagram were used on 84 osteosarcoma samples from the TARGET database (https://xena.ucsc.edu/, accessed on 6 April 2023) to verify this relationship based on the R packages “ROCR” and “ggplot2.” The expression level of BNIP3 in each tumor was studied using GEPIA.

### 2.8. Enrichment Analysis of Biologically Relevant Pathways

Then, we screened the DEGs among different risk subgroups using the R package “DESeq2” with the following limiting condition: log2|folding change| > 1 and adjusted *p* value < 0.05. The database pathways of GO and Kyoto Encyclopedia of Genes and Genomes (KEGG) were explored to elucidate biologically relevant pathways using the R packages “clusterProfiler” (version 4.6.2), “org.Hs.eg.db” (version 3.16.0), and “enrichplot” (version 1.18.4).

### 2.9. Exploration of Immune Microenvironment Landscape

To explore the association between DGPS and the infiltration of immune microenvironment landscape, a single-sample gene set enrichment analysis (ssGSEA) algorithm in the R package “GSVA” was then used on 84 osteosarcoma samples from the TARGET database (https://xena.ucsc.edu/, accessed on 6 April 2023) to evaluate the infiltration and functional scoring of immune cells in osteosarcoma. Immune checkpoint correlation analysis was carried out using the R package “limma” (version 3.54.2).

### 2.10. Exploration of Drug Sensitivity

The sensitivity to chemotherapeutic drugs was represented by the half-maximal inhibitory concentration (IC50) of chemotherapeutic drugs. IC50 is a crucial indicator to access tumor response to therapy; a smaller IC50 indicates a higher sensitivity of tumor cells to this chemotherapeutic agent, and the opposite indicates a lower sensitivity. In order to assess the value of DGPS in the clinical management of OS and find suitable potential chemotherapeutic drugs for patients with osteosarcoma, the drug therapy response was evaluated for each patient using the R package “pRRophetic” (version 6) (the drug source was the 251 anticancer drugs in the R package “pRRophetic” (version 6)). This was accomplished by creating statistical models of gene expression and drug sensitivity data from cell lines in the Cancer Genome Project (CGP) and then applying these models to the oncogene expression levels in tumor samples to generate in vivo drug sensitivity predictions [15]. The IC50 values of different subgroups were then compared using the Wilcoxon signed-rank test.

### 2.11. Cell Culture

The human osteosarcoma cell line 143B (CRL-8303) was purchased from American Type Culture Collection (ATCC) and was cultured in Dulbecco’s Modified Eagle’s Medium (DMEM) under conditions of 5% CO_2_ at 37 °C. The medium was supplemented with 10% fetal bovine serum (FBS), 100 IU/mL penicillin, and 100 mg/mL streptomycin [16].

### 2.12. Apoptosis Analysis by Flow Cytometry

Flow cytometry was used to detect cell apoptosis. The cells were washed twice with precooled PBS. Follow-up assays were carried out according to the instructions of the apoptosis kit. The collected cells were resuspended in a 10 mL centrifuge tube containing a 1× binding buffer to make 1 × 10^6^ cells/mL. 5 μL Annexin V/PI was used for staining at room temperature for 15 min. Eventually, 400 μL 1× Binding Buffer was added to detect apoptosis of 143B cells by flow cytometry.

### 2.13. 5-Ethynyl-2′-Deoxyuridine (EdU) Experiment

The EyoClick EdU Cell Proliferation Kit with Alexa Fluor 594 was used to verify the proliferation ability of cells. 143B cells with BNP3 knocked out or overexpressed were seeded at a density of 5 × 10^4^ cells/well in a 6-well plate covered with 24 mm × 24 mm coverslips and synchronized with pure medium for 12 h. The medium was completely replaced with MEM medium. The kit’s instructions were followed for washing and fixation. The 143B cells were stained with apollo fluorescent board azide 594 under CuSO_4_ for 30 min at 37 °C. After that, cell nuclei were stained with DAPI (10 μg/mL) for 10 min and washed with PBST to completely remove the unbound DAPI, the cover was removed, and the glass was secured. Fluorescence microscopy was used to observe the fluorescence staining of cells (Olympus, Japan).

### 2.14. Wound-Healing Assay

Osteosarcoma cells of different transfection types (4 × 10^5^) were cultured on 6-well plates for 12 h to ensure 80% cell density was achieved. Artificial wounds were made using the tip of 200 μL pipette, and phosphate-buffered saline was used to wash the nutrient solution twice. Finally, 3~5 mL of the serum-free nutrient solution was added and the migration distance was measured at 0 and 24 h after injury.

### 2.15. Statistical Analysis

All statistical analysis was carried out using R software (version 4.1.2). RNA-seq transcriptome data were included in the TARGET (https://xena.ucsc.edu/, accessed on 6 April 2023) and GEO (https://www.ncbi.nlm.nih.gov/geo/, accessed on 6 April 2023) databases. The Wilcoxon rank sum test was applied to compare the differences between different risk subgroups of quantitative data. The criterion for a statistically significant difference was set at *p* < 0.05.

## 3. Results

### 3.1. Identification of Prognosis Related-DRGs

**Figure 1:**
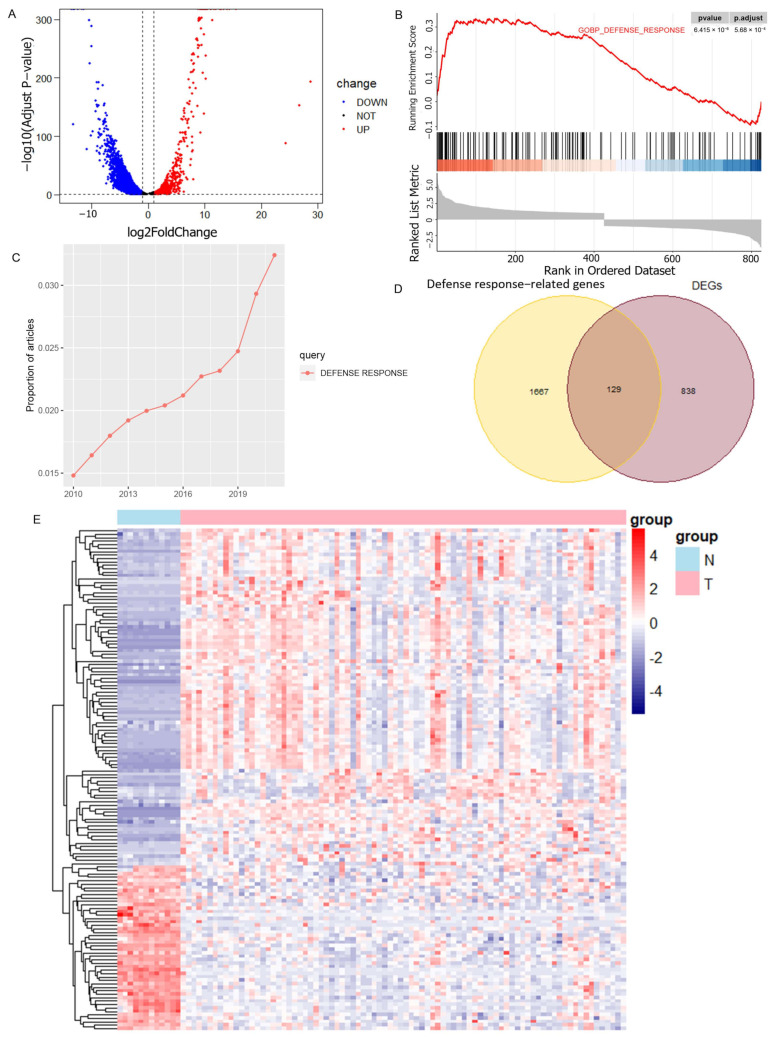
Screening of DEGs related to the defense response. (**A**) Volcano plot illustrating the DEGs in osteosarcoma and normal groups with the threshold set at |logFC| ≥ 1 and adj-*p* ≤ 0.05. (**B**) DEGs are significantly enriched in the GOBP_DEFENSE_RESPONSE pathway. (**C**) Trend in the number of studies on GOBP_DEFENSE_RESPONSE pathways in recent years. (**D**) DRGs obtained by taking the intersection of DEGs and GOBP_DEFENSE_RESPONSE pathway genes. (**E**) Heatmap showing the expression of DRGs in osteosarcoma samples and normal samples.

Differential gene analysis was performed using 12 normal samples and 84 osteosarcoma samples from the GSE33383 dataset as a method to obtain the differentially expressed genes in osteosarcoma (Figure 1A), and GSEA analysis showed that these genes were significantly enriched in the GOBP_DEFENSE_RESPONSE pathway (Figure 1B). From the search results of the relevant literature in PubMed Central, it was found that the number of studies on the GOBP_DEFENSE_RESPONSE pathway in the last 10 years demonstrated a significantly increasing trend (Figure 1C). In total, 129 DRGs were obtained from the intersection of the gene set of this pathway and the osteosarcoma DEGs (Figure 1D). Their expression in osteosarcoma and normal samples can be seen in the heatmap (Figure 1E).

### 3.2. DGPS Was Validated as an Independent Prognostic Factor of Osteosarcoma

**Figure 2:**
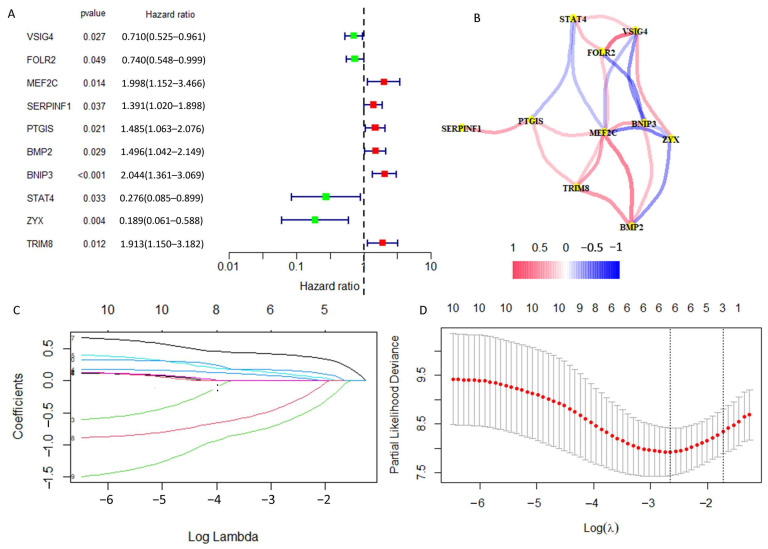
Obtaining DRGs associated with osteosarcoma prognosis. (**A**) Univariate Cox regression analysis for identifying prognostic DRGs. (**B**) Interaction network diagram of prognosis-related DRGs. (**C**,**D**) Lasso–Cox regression analysis was performed to construct prognostic prediction models.

**Figure 3:**
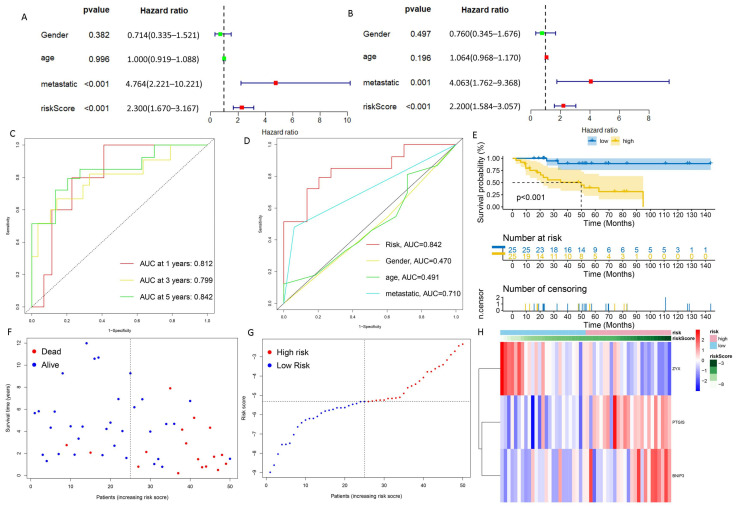
Evaluation of DGPS. (**A**) Univariate Cox analysis. Risk score and metastasis were statistically significant. (**B**) Multivariate Cox analysis. (**C**) ROC curve of DGPS in training group. (**D**) ROC demonstrating that the predictive accuracy of DGPS was superior to the other clinical parameters in the training set. (**E**) Kaplan–Meier curves of overall survival in the training set. (**F**,**G**) Distribution of risk scores and distribution of overall survival status and risk score in the training set. Blue: low risk; red: high risk. (**H**) Heatmap indicating the expression degrees of BNIP3, PTGIS, and ZYX in the training set. ROC curve, receiver operating characteristics curve; AUC, area under the curve; *p* < 0.05, statistically significant.

**Figure 4:**
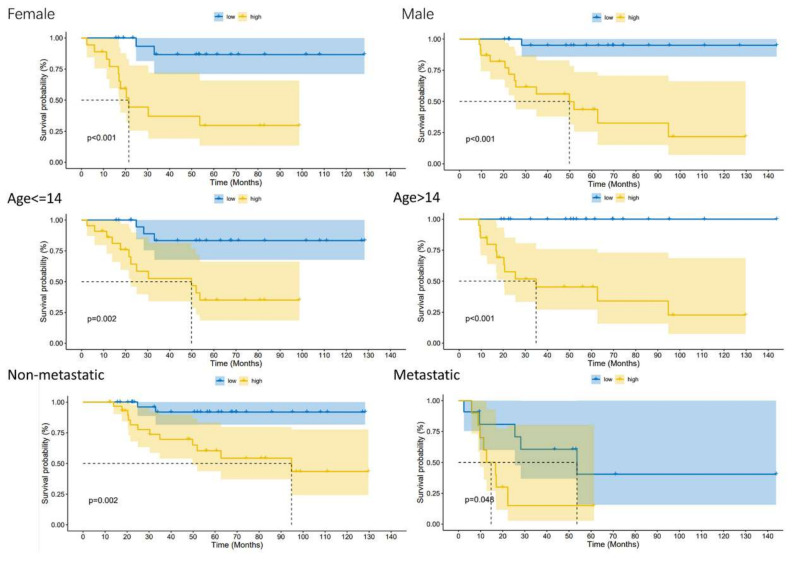
Kaplan–Meier plots depicting subgroup survival analyses stratified by gender, age, and metastasis.

We carried out univariate Cox regression analysis on these 129 DRGs and obtained 10 DRGs with prognostic value (Figure 2A). The correlation network map (Rcutoff = 0.2) found BNIP3 and ZYX to be at the center of 10 risk DRGs (Figure 2B). The 10 risk DRGs were selected by LASSO analysis (Figure 2C,D) before multivariate Cox analysis was executed, then we screened three DRGs to establish DGPS: RiskScore = 0.42* Expression PTGIS + 0.567* Expression BNIP3 − 1.477* Expression ZYX. We then distributed patients into different risk subgroups based on the median risk score. We carried out univariate and multivariate Cox regression analyses on risk scores and clinical characteristics as a means of assessing the predictive value of DGPS. Then, statistically significant differences were observed for risk score and metastasis (Figure 3A). Meanwhile, risk score and metastasis retained prognostic value for OS in multivariate Cox regression analysis (Figure 3B). The AUC values of the one-year, three-year, and five-year ROC curves reached 0.812, 0.799, and 0.842, respectively, showing accurate prediction of the survival rate of osteosarcoma patients at one, three, and five years by DGPS (Figure 3C). The ROC plots of DGPS and the clinical characteristics showed that the AUC values of DGPS were higher than those of all the other clinical characteristics (Figure 3D). The Kaplan–Meier survival curve showed significant differences in survival status among patients in different risk subgroups based on DGPS division (Figure 3E). The risk score distribution and survival status maps showed that among patients classified into different risk subgroups according to the median risk score, mortality was higher in the high-risk group (Figure 3F,G). The risk heatmap showed that BNIP3 and PTGIS were highly expressed in the high-risk group, while ZYX was highly expressed in the low-risk group (Figure 3H). A stratified subgroup analysis was then performed as a means of exploring the prognostic value of DGPS among all cohorts. We divided the entire cohort into various sets by gender, age (<14 and ≥14), and metastasis. Based on DGPS among different clinical groups, the KM survival curve indicated that the low-risk group had significantly higher overall survival compared to the high-risk group (Figure 4). These findings all demonstrated that DGPS could be an independent prognostic factor for use in the accurate prediction of OS patient prognosis.

### 3.3. Verification of DGPS

**Figure 5:**
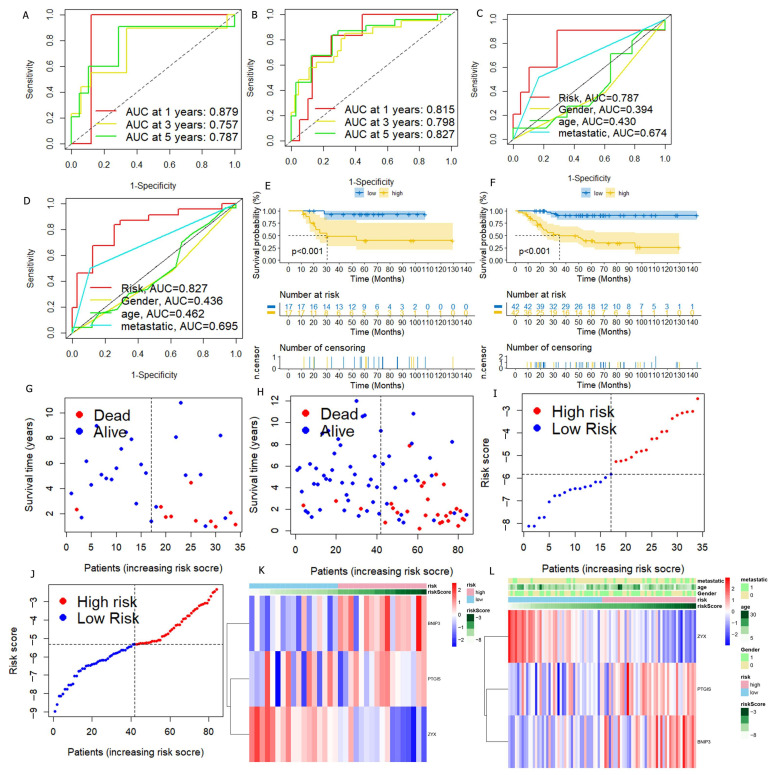
Verification of DGPS. ROC curve of DGPS in the test set (**A**) and in the entire cohort (**B**). ROC demonstrated that the predictive accuracy of DGPS was superior to that of other clinical characteristics in the test set (**C**) and in the entire cohort (**D**). Kaplan–Meier curves of overall survival (OS) in the test set (**E**) and in the entire cohort (**F**). Survival status of patients with osteosarcoma in the test set (**G**,**I**) and in the entire cohort (**H**,**J**). Blue: low risk; red: high risk. The heatmap indicates the expression degrees of BNIP3, PTGIS, and ZYX in the test set (**K**) and in the entire cohort (**L**).

**Figure 6:**
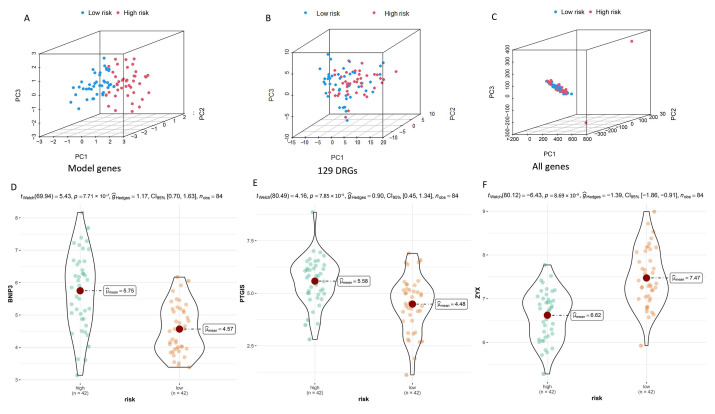
PCA plots depicting the distribution of samples based on the expression of model genes (**A**), DRGs (**B**), and all genes (**C**). Differential expression of model genes in the high- and low-risk groups is shown in box plots (**D**–**F**).

In order to verify the stability of DGPS, the test set and all cohorts were investigated using ROC, Kaplan–Meier survival curves, risk score distribution, survival status maps, and risk heatmaps. The AUC at one, three, and five years reached 0.879, 0.757, and 0.787 in the test set, respectively, and reached 0.815, 0.798, and 0.827 in the entire cohort, respectively (Figure 5A,B). The AUC value of DGPS was higher than that of the other clinical characteristics in the test set and all cohorts (Figure 5C,D). The KM survival curves of the test set and entire cohort showed that the low-risk group had a higher overall survival rate than the high-risk group (Figure 5E,F). The distribution of risk scores and the distribution of overall survival status and risk score were verified in the test set and all cohorts. (Figure 5G–J). The risk heatmaps of the test set and all cohorts showed that the risk genes were expressed at the same level in the high- and low-risk subgroups as in the training set (Figure 5K,L). PCA was carried out to explore the differences between different risk subgroups. DGPS divided different risk subgroups into two clusters, but there was no significant distinction based on the expression of 129 DRGs and all genes (Figure 6A–C). The box diagrams showed that the expression of BNIP3 and PTGIS was significantly lower in the low-risk group than in the high-risk group, while the opposite was true for the expression of ZYX (Figure 6D–F).

### 3.4. Construction and Verification of Nomogram

**Figure 7:**
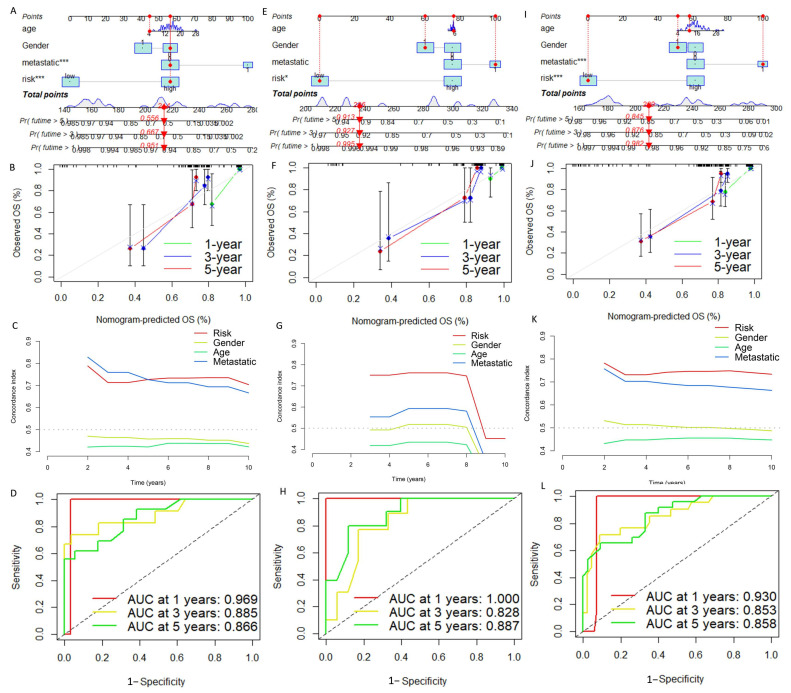
Construction and evaluation of a nomogram based on DGPS. Nomogram used to predict prognosis was constructed based on DGPS in the training set (**A**), test set (**E**), and entire cohort (**I**). Calibration curves of the nomogram in the training set (**B**), test set (**F**), and entire cohort (**J**). The C-index curves for assessing the discrimination ability of DGPS and other clinical characteristics at each time point in the training set (**C**), test set (**G**), and entire cohort (**K**). ROC curves of the nomograms at one, three, and five years in the training set (**D**), test set (**H**), and entire cohort (**L**). “*”represented “*p* < 0.05”, “***”represented “*p* < 0.001”.

We constructed a nomogram to precisely predict the one-, three-, and five-year survival probability of OS (Figure 7A). The calibration curves indicated that the one-, three-, and five-year survival rates calculated by the nomogram were satisfactorily consistent with the actual OS patient survival rate (Figure 7B). In addition, the C-index indicated that the predictive accuracy of DGPS was better than that of other clinical features (Figure 7C). ROC curves constructed based on the nomogram at one, three, and five years showed that the nomogram had excellent prognostic predictive value (Figure 7D). The same tests showed good performance for both the test set and all cohorts (Figure 7E–L).

### 3.5. Exploration of the Association of Tumor Metastasis with BNIP3

**Figure 8:**
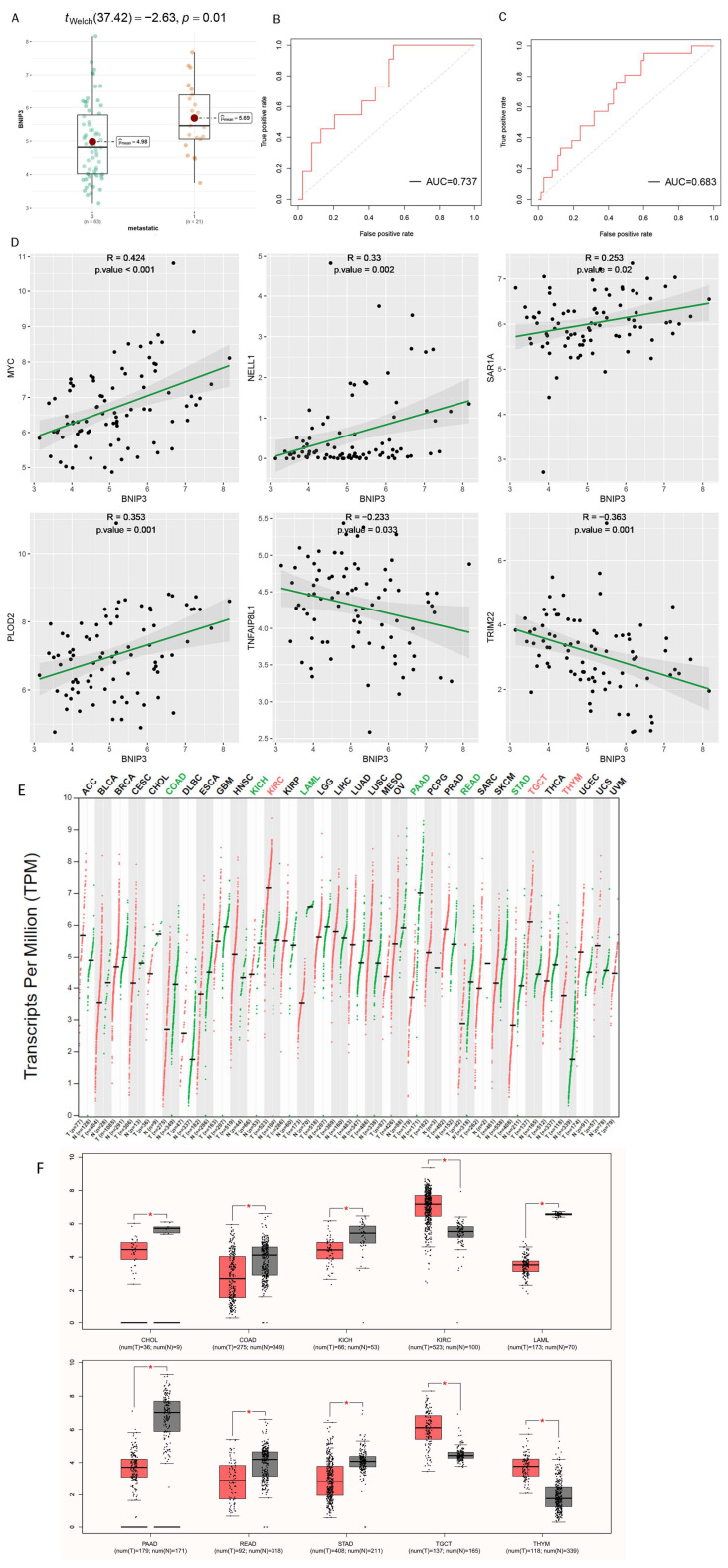
Exploration of the association of tumor metastasis with BNIP3. (**A**) Correlations between BNIP3 and osteosarcoma metastasis are displayed in box plots. ROC curve of diagnosis of osteosarcoma metastasis by BNIP3 in the training set (**B**) and in the entire cohort (**C**). (**D**) Relationship between the expression of BNIP3 and the expression of tumor metastasis-related genes MYC, NELL1, SAR1A, PLOD2, TNFAIP8L1, and TRIM22. (**E**,**F**) Expression of BNIP3 in different tumors. “*”represented “*p* < 0.05”.

**Figure 9:**
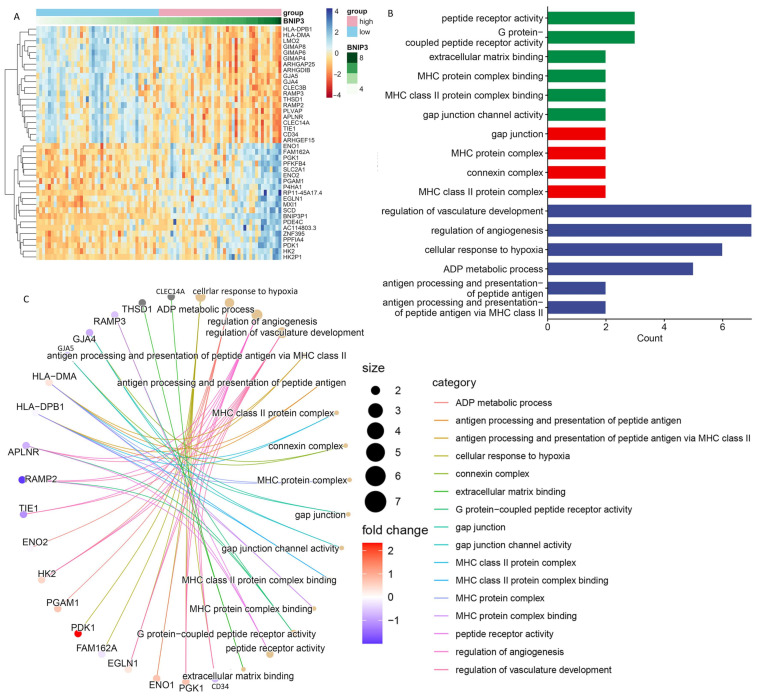
Pathway enrichment analysis of the genes most strongly associated with BNIP3. (**A**) Heatmap showing the 20 genes with the strongest positive or negative correlations with BNIP3 expression. (**B**,**C**) Pathway enrichment analysis showing the enrichment of genes in different pathways.

The expression of BNIP3 was detected in different metastatic subgroups. The box diagram results demonstrated that the expression of BNIP3 was significantly lower in the non-metastatic group than in the metastatic group (Figure 8A). The ROC curve in the training set demonstrated that the expression of BNIP3 had satisfactory accuracy for the prediction of tumor metastasis (Figure 8B), which was also verified in all cohorts (Figure 8C). In addition, the relationship between BNIP3 expression and OS metastasis-related gene expression can be seen in Figure 8D. BNIP3 was positively correlated with genes that promote osteosarcoma metastasis, such as MYC, NELL1, SAR1A, and PLOD2, and negatively correlated with genes that inhibit osteosarcoma metastasis, such as TNFAIP8L1 and TRIM22, which suggested that a certain association exists between BNIP3 expression and OS metastasis. Pan-cancer analysis performed in the GEPIA database showed that BNIP3 was lowly expressed in tumors such as CHOL and COAD and highly expressed in tumors such as KIRC and TGCT (Figure 8E,F). The top 20 genes with the strongest positive or negative correlations with BNIP3 expression were further screened for enrichment analysis, and the results showed that these genes were significantly enriched in pathways such as antigen processing and presentation of peptide antigen, regulation of angiogenesis, cellular response to hypoxia, gap junction channel activity, etc. (Figure 9A–C).

### 3.6. Enrichment Analysis of Biologically Relevant Pathways

**Figure 10:**
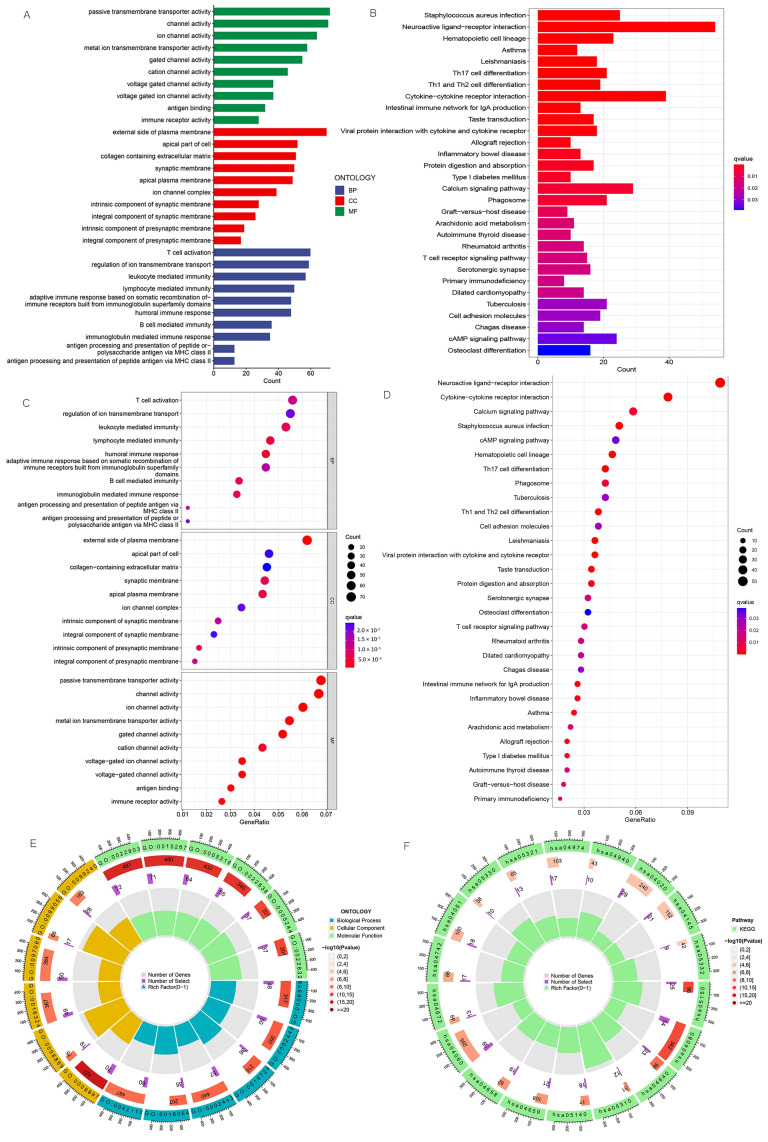
GO and KEGG pathway enrichment analyses. (**A**) Bar plot of the top 10 GO enrichment terms. (**B**) Bar plot of the top 30 KEGG enrichment terms. (**C**) Bubble chart of the top 10 GO enrichment terms. (**D**) Bubble chart of the top 30 KEGG enrichment terms. (**E**) Circle diagram of GO enrichment analysis. (**F**) Circle diagram of KEGG enrichment analysis. GO enrichment terms include biological process, cellular component, and molecular function.

We carried out GO and KEGG enrichment analyses to explore the biologically relevant functions and pathways between different risk subgroups, and we identified 3206 DEGs. In the biological process category, the genes were mainly enriched in T cell activation, B cell-mediated immunity, and leukocyte-mediated immunity. In the cellular component category, they were found to be mainly enriched in the external side of the plasma membrane, the apical part of the cell, etc. In the molecular function category, they were found to be primarily enriched in antigen binding, immune receptor activity, and passive transmembrane transporter activity (Figure 10A,C,E). KEGG enrichment analysis demonstrated that DEGs were primarily enriched in Th17 cell differentiation, Th1 and Th2 cell differentiation, the T cell receptor signaling pathway, etc. (Figure 10B,D,F).

### 3.7. Exploration of Relationship between Immune Microenvironment and DGPS

**Figure 11:**
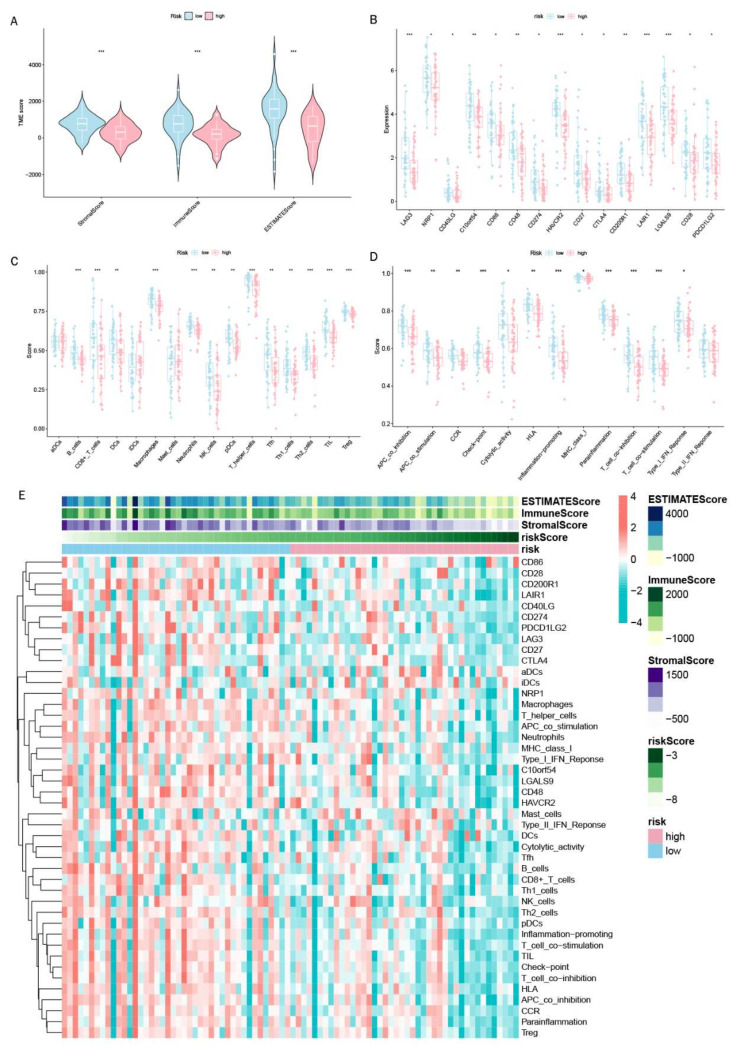
Immunoassay showing that DGPS is closely related to the immune system. (**A**) Analysis of TMB differences between high- and low-risk groups of patients with osteosarcoma. Box plots of the ssGSEA scores of 15 immune checkpoints (**B**), 13 immune cells (**C**), and 12 immune-related functions (**D**) between different risk groups. (**E**) Heatmap showing the landscape of immune characteristics and the tumor microenvironment in the TARGET cohort determined by the ssGSEA algorithm. “*”represented “*p* < 0.05”,“**”represented “*p* < 0.01”,“***”represented “*p* < 0.001”.

**Figure 12:**
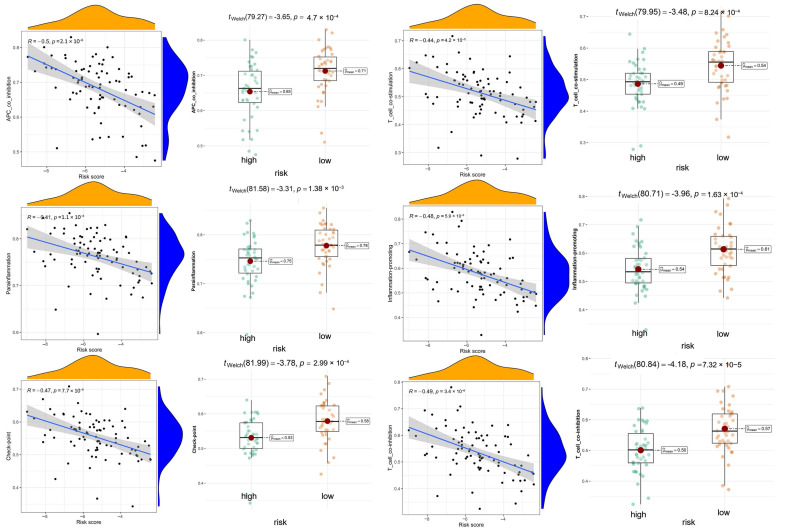
The association between immune functions and risk scores and immune function scores between different risk subgroups.

**Figure 13:**
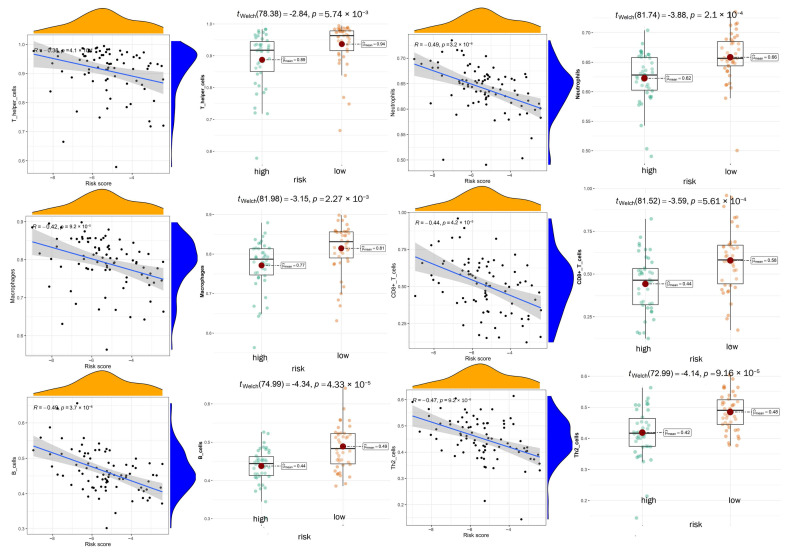
The association between immune cells and risk scores and immune cell scores between different risk subgroups.

**Figure 14:**
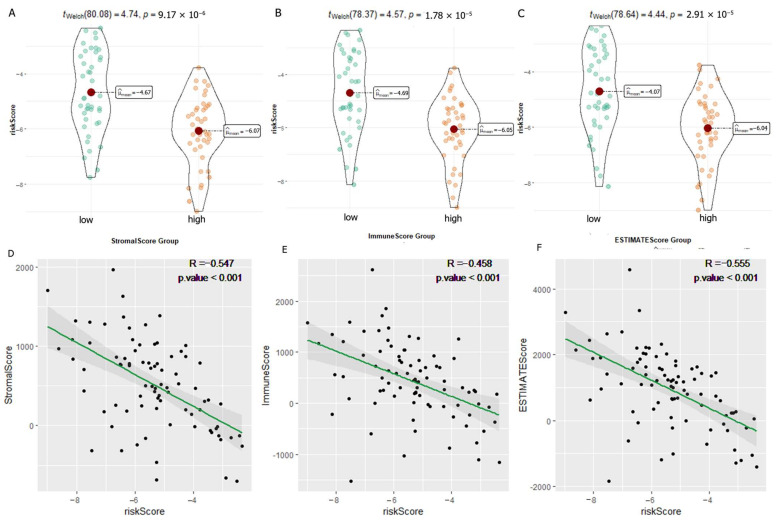
Correlation analysis of immune-related scores and risk scores. (**A**–**C**) Analysis of the variability of risk scores among different StromalScore (**A**), ImmuneScore (**B**), and ESTIMATEScore (**C**) subgroups. (**D**–**F**) Scatter plots of correlations between risk scores and StromalScore (**D**), ImmuneScore (**E**), and ESTIMATEScore (**F**).

We carried out multiple immune assessment algorithms as a means of investigating the difference in the TME landscape of OS patients in different risk subgroups. From the ESTIMATE results, we found that patients in the low-risk group had higher stromal, immune, and ESTIMATE scores and lower tumor purity than the high-risk group (Figure 11A). We found that DGPS was associated with immune checkpoint-related gene expression, with the high-risk group showing high levels of expression of LAG3, CD274, CD27, CTLA4, etc. (Figure 11B). In addition, the immune cell differential analysis demonstrated that B cells, CD8+T cells, neutrophils, NK cells, pDCs, Th1 cells, Th2 cells, and TILs were significantly downregulated in the high-risk group (Figure 11C). Immune function analysis found that the low-risk group had higher immune function scores in categories such as CCR, checkpoint, T cell co-inhibition, etc. than the high-risk group (Figure 11D). The levels of immune characteristics were higher in the low-risk group than in the high-risk group (Figure 11E). The relationship between immune cells (Figure 12) and immune functions (Figure 13) with risk score and different risk subgroups was also explored. These results all indicated that DGPS had an association with the immune microenvironment and could reveal OS patient immunity status (Figure 14).

### 3.8. Anticancer Drug Sensitivity Analysis

**Figure 15:**
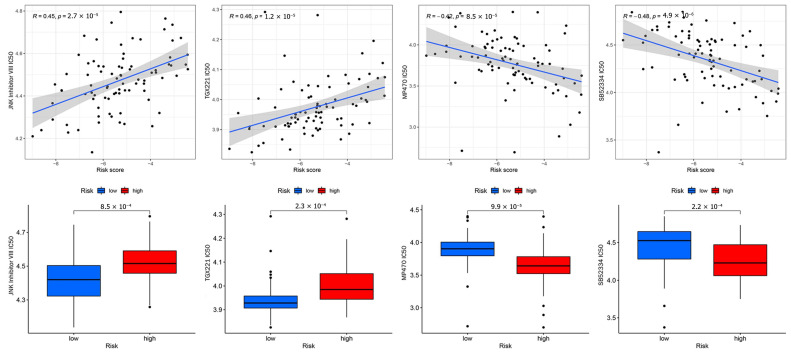
Drug correlation and sensitivity analyses with JNK Inhibitor VIII, TGX221, MP470, and SB52334.

Targeted drug therapy is a crucial strategy in tumor therapy. We performed drug sensitivity analysis with 251 anticancer drugs using the R package “pRRophetic” (version 6). This was achieved by creating statistical models from gene expression and drug sensitivity data from cell lines in the Cancer Genome Project (CGP) and then applying these models to oncogene expression levels in tumor samples in order to generate in vivo drug sensitivity predictions. To determine the potential use of DGPS, we investigated the association between DGPS and drug IC50 in OS therapy by comparing the differences in anticancer drug sensitivity between the two different risk groups. The IC50 values of anticancer drugs were compared in the different risk subgroups. The IC50 values of four anticancer drugs were found to be statistically different between the different risk subgroups (*p* < 0.05), as can be seen in Figure 15. The IC50 values of MP470 and SB52334 were lower in the high-risk group, proving that the high-risk group had greater sensitivity to these drugs. In addition, the IC50 values of JNK Inhibitor VIII and TGX221 were lower in the low-risk group, indicating that the low-risk group had greater sensitivity to these drugs. This finding suggested that the new DGPS signal may be helpful in predicting the efficacy of chemotherapy in patients with osteosarcoma.

### 3.9. BNIP3 Regulates Apoptosis in Osteosarcoma Cells

**Figure 16:**
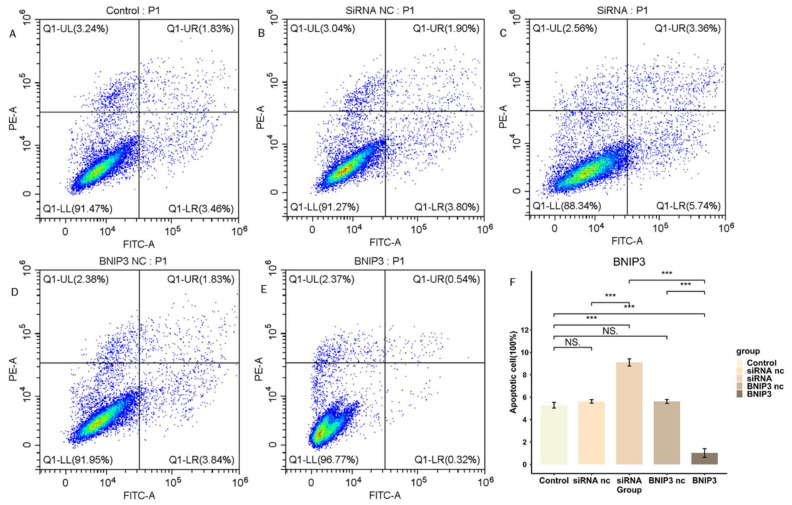
BNIP3 regulates the apoptosis of osteosarcoma cells. (**A**–**E**) Apoptosis of osteosarcoma cells after knockdown or overexpression of BNIP3. (**F**) Overexpression of BNIP3 inhibits apoptosis of osteosarcoma cells, while knockdown of BNIP3 promotes apoptosis of osteosarcoma cells. “NS” represented “No significant difference”, “***” represented “*p* < 0.001”.

We investigated the regulation of apoptosis in osteosarcoma cell line 143B by BNIP3 expression levels. We knocked down BNIP3 expression in 143B cells by siRNA transfection and overexpressed BNIP3 in 143B cells by adenoviral transfection, and the apoptosis rate of cells was detected by flow cytometry (Figure 16). The results demonstrated that knockdown of BNIP3 significantly elevated the apoptosis rate of osteosarcoma cells, while overexpression of BNIP3 significantly decreased the apoptosis rate of osteosarcoma cells. In conclusion, the experimental results confirmed that the expression level of BNIP3 played an apoptosis-regulating role in osteosarcoma cell line 143B.

### 3.10. BNIP3 Promotes Osteosarcoma Progression

**Figure 17:**
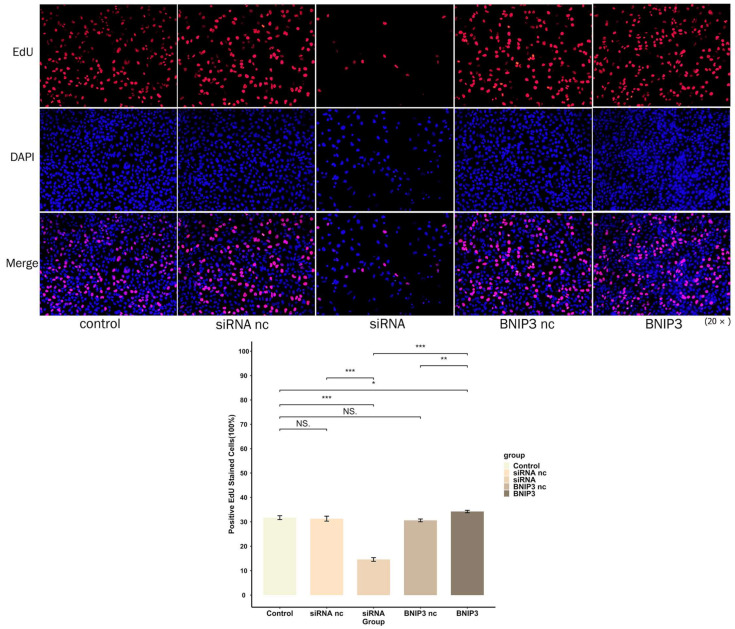
BNIP3 regulates the proliferation of osteosarcoma cells. Knockdown of BNIP3 inhibits the proliferation of osteosarcoma cells, while overexpression of BNIP3 promotes the proliferation of osteosarcoma cells. “NS” represented “No significant difference”, “*” represented “*p* < 0.05”, “**” represented “*p* < 0.01”, “***” represented “*p* < 0.001”.

**Figure 18:**
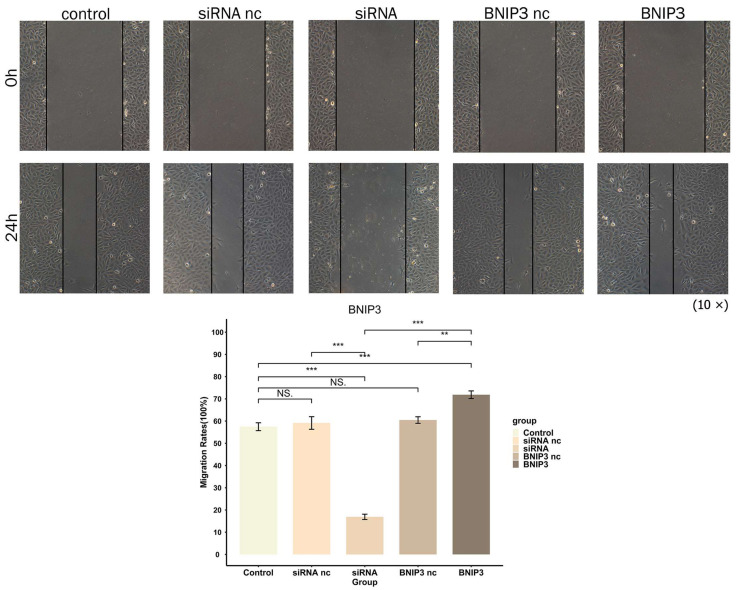
BNIP3 regulates the migration ability of osteosarcoma cells; knockdown of BNIP3 inhibits their migration ability, while overexpression of BNIP3 promotes the migration ability of osteosarcoma cells. “NS” represented “No significant difference”, “**” represented “*p* < 0.01”, “***” represented “*p* < 0.001”.

The proliferation of 143B cells was detected by EdU experiments. As shown in Figure 17, the proliferation of osteosarcoma cells was significantly inhibited after knockdown of BNIP3, while overexpression of BNIP3 significantly increased the proliferation of osteosarcoma cells. The wound healing assay was used to detect the migration ability of osteosarcoma cells, the results of which can be seen in Figure 18. Osteosarcoma cell migration significantly decreased following knockdown of BNIP3, while it was significantly enhanced following BNIP3 overexpression. These results confirmed the promoting effect of BNIP3 on osteosarcoma progression and metastasis.

## 4. Discussion

Tumor formation is a complex, multi-stage process in which human cells must break through multiple lines of defense before becoming tumor cells. Osteosarcoma is the most common primary malignant tumor of bone in children and young adults, which is characterized by easy recurrence, strong invasiveness, and early metastasis, and it is a tumor with a high degree of malignancy. Combination chemotherapy and complete surgical resection of osteosarcoma is key to a cure, but this only applies to localized osteosarcoma and primary metastatic osteosarcoma. Surgery requires removal of all known metastatic deposits [17], but osteosarcoma patients are prone to lung metastasis. The five-year survival rate for patients with metastatic osteosarcoma remains low, so predicting tumor metastasis and identifying new prognostic biomarkers are essential.

A potential relationship may exist between genes related to the defense response and tumor occurrence and development. In the process of tumor occurrence, the body initiates a series of defense responses, including cellular immune defense, humoral immune defense, and producing cytokines as a response to DNA damage events, all of which are important host defense responses [2,12]. The link between cancer and defense response genes has been identified by several previous studies, including key prognostic genes of osteosarcoma [4], pancreatic cancer [5] clear cell renal cell carcinoma [6], and breast cancer [7] that are significantly enriched in the defense response pathway. Defense response production helps the body to kill tumor cells while preventing their escape [8,9,10]. Therefore, defense response-related genes have a close relationship with osteosarcoma development and progression. Relatively few studies have been conducted on the role that defense response plays in osteosarcoma. This study established a signature composed of three genes for predicting osteosarcoma prognosis from a defense response perspective for the first time. The model was constructed using a GEO dataset and the TARGET database. It demonstrated good predictive performance for the overall survival of osteosarcoma patients and served as an independent prognosis predictor for osteosarcoma patients in both the training and test sets divided from the TARGET database. In addition, it exhibited excellent performance in distinguishing high- and low-risk groups. A new nomogram was produced for the purpose of guiding osteosarcoma treatment. Three key genes were studied to varying degrees, and BNIP3 was found to play an essential role in osteosarcoma metastasis and progression, verified by apoptosis, proliferation, and migration experiments. Apoptosis and EdU experiments confirmed that BNIP3 can inhibit osteosarcoma cell apoptosis and promote proliferation. Migration experiments confirmed that as a risk gene, BNIP3 can enhance osteosarcoma cell invasiveness and promote metastasis. Box plots and ROC curves proved that BNIP3 had good performance in osteosarcoma metastasis prediction. Further correlation analysis found BNIP3 to have a significantly positive correlation with MYC, NELL1, SAR1A, PLOD2, and other genes proven to promote osteosarcoma metastasis [18,19,20,21]. However, a significantly negative correlation was found between TNFAIP8L1, TRIM22, and other genes proven to inhibit osteosarcoma metastasis [22,23]. In addition, pan-cancer analysis revealed that BNIP3 was also differentially expressed in different tumor tissues compared to that in normal tissues, including LAML, COAD, and KIRC, which was consistent with previous studies [24,25,26,27]. Enrichment analysis was conducted on the differentially expressed genes of the BNIP3 high–low expression group. The results indicated that the differentially expressed genes were significantly involved in antigen processing and the presentation of peptide antigen, the regulation of angiogenesis and cellular response to antigen hypoxia, gap junction channel activity, and other pathways, suggesting that BNIP3 may facilitate the regulation of osteosarcoma progression via these pathways, which needs to be explored in further experiments. All of these results suggested that BNIP3 has the potential to be a therapeutic target for osteosarcoma, with implications for clinical treatment and future research in osteosarcoma.

Among the three identified genes, BNIP3 has been reported to have an association with osteosarcoma, ovarian cancer, breast cancer, and melanoma prognosis [28,29,30,31], and BNIP3 is pro-apoptotic in most studies [32], although there are still some studies indicating that BNIP3 can inhibit apoptosis in tumor cells [33,34,35]. Burton et al. [35] reported that nuclear BNIP3 acts as a transcriptional repressor by binding to the promoter region of the AIF gene, thereby preventing apoptosis of glioma cells. Luo et al. [33] indicated that knockdown of BNIP3 significantly increased the apoptosis rate of lung cancer cells, and low expression levels of BNIP3 could increase the infiltration of immune cells and improve the prognosis of lung cancer patients. Moreover, the role of BNIP3 in various tumors is inconsistent. Vianello et al. [29] found that high levels of BNIP3 expression significantly reduced survival in ovarian cancer patients, and that BNIP3 affected tumor cell resistance by regulating mitochondrial autophagy and could also be a potential target for new therapeutic strategies. Niu et al. [30] discovered BNIP3 as a tumor suppressor by alleviating FTO-dependent breast tumor growth and metastasis. Hu et al. [36] found that BNIP3 could serve as a prognostic biomarker for breast cancer patients, and patients with breast cancer with high BNIP3 expression had poorer overall survival, disease-free survival (DFS, the measure of time after treatment during which no sign of cancer is found [37]), and disease-specific survival (DSS, the percentage of people who die from a specific disease in a defined period of time, i.e., patients who die from causes other than the disease being studied are not counted [38]). These studies have fully illustrated the diversity of BNIP3 modes of action and biological functions under different conditions.

ZYX has been reported to have an association with glioblastoma, colon cancer, and pancreatic cancer [39,40,41,42]. However, its role in various tumors is also not standardized, and studies on its role in osteosarcoma are lacking. Michiyo et al. [43] found that inhibition of ZYX expression could lead to tumor regression by affecting cell structure and motility in oral squamous cell carcinoma cells, while Aleksandra et al. [44] indicated that decreased expression of ZYX may promote the formation of non-small cell lung cancer. The role of PTGIS in osteosarcoma has not yet been reported, but some studies have demonstrated that PTGIS is associated with the prognosis of bladder and lung cancers [45,46,47]. Kai et al. [48] revealed that PTGIS promotes proliferation, migration, and invasion of lung squamous cell carcinoma (LUSC) and can be utilized as a therapeutic target for LUSC as well as a biomarker for prognosis and tumor immunity. Danian et al. [49] found that high expression of PTGIS promoted the infiltration of tumor-associated macrophages (TAMs) and Tregs in the tumor microenvironment and deteriorated the prognosis of patients with lung, ovarian, and gastric cancers.

Immunotherapy has been developed as a complementary treatment strategy to conventional chemotherapy. To find the application value of DGPS in the immunotherapy of osteosarcoma, we further explored the tumor microenvironment of osteosarcoma in our present study. We performed differential analysis of the tumor microenvironment in the high- and low-risk groups classified based on DGPS and found that immune infiltration was significantly higher in the low-risk group than in the high-risk group. The scores of most immune cells in the low-risk group in this study were found to be significantly higher than those of the high-risk group, such as B cells, macrophages, neutrophils, NK cells, CD8+ T cells, Th1 cells, and Th2 cells. Meanwhile, the enrichment analysis of DEGs in the different risk subgroups also revealed significant enrichment in KEGG enrichment pathways such as Th1 and Th2 cell differentiation and in GO enrichment pathways such as B cell-mediated immunity and T cell activation. The immune function scores of APC co-inhibition, CCR, checkpoint, cytolytic activity, and T cell co-stimulation were also found to be significantly higher in the low-risk group. This may serve as a basis for selecting appropriate therapeutic targets and chemotherapeutic agents. In addition, the low-risk group had higher stromal, immune, and ESTIMATE scores, which indicated lower tumor purity and better immunotherapy response among patients in the low-risk group [50,51]. Additionally, we also divided all samples into high and low ImmuneScore groups according to the median ImmuneScore for the difference analysis of DGPS risk scores, and the results indicated that the low ImmuneScore group had a significantly higher DGPS risk score than the high ImmuneScore group, with the same results validated for StromalScore and ESTIMATEScore. Correlation scatterplots indicated that DGPS risk scores were significantly and negatively correlated with ImmuneScore, StromalScore, and ESTIMATEScore. These results suggested that osteosarcoma in the high-risk group of DGPS can be classified as a cold tumor subgroup, i.e., with poorer immune infiltration and possibly poorer response to immunotherapy [52,53]. The expression of immune checkpoints LAG3, NRP1, CD40LG, C10orf54, CD86, CD48, CD274, HAVCR2, CD27, CTLA4, CD200R1, LAIR1, LGALS9, CD28, and PDCD1LG2 was significantly higher in the low-risk group than in the high-risk group, and it was previously confirmed in the literature that LAG3 [54], CD86 [55], HAVCR2 [56], CD27 [57], LAIR1 [58], and PDCD1LG2 [56] checkpoints play important roles in osteosarcoma cell therapy. These results suggested that these checkpoints could be potential targets for osteosarcoma therapy and that DGPS could provide a new criteria to guide osteosarcoma immunotherapy.

The sensitivity to chemotherapeutic drugs was represented by the half-maximal inhibitory concentration (IC50) of chemotherapeutic drugs. IC50 is a crucial indicator for assessing tumor response to therapy; a smaller IC50 value indicates higher sensitivity of tumor cells to this chemotherapeutic agent. IC50 values have been used by many researchers to predict drug sensitivity in order to explore personalized drug therapy guidance for different patients [59,60,61,62,63,64]. At the same time, high-risk patients in this study were found to have a greater sensitivity to MP479, SB52334, and other drugs, whereas low-risk patients were found to be more sensitive to JNK inhibitor VIII and TGX221. It was previously found that MP470 could be a potential therapeutic agent for osteosarcoma [65]. The results showed that DGPS has great potential for guiding clinical treatment strategies for osteosarcoma patients.

There were several limitations to this study. Firstly, the datasets were downloaded from the TARGET and GEO databases, and the sample quantities were limited. Secondly, external validation of the constructed model was not conducted to improve its applicability. Generally, the model had good prognostic value, and the role played by the BNIP3 gene in osteosarcoma occurrence and development was verified experimentally. At the same time, collecting more clinical samples is planned for further verification of this model.

From this study, it can be seen that the signature based on the defense response has good application value for the prediction of osteosarcoma prognosis, and it has strong potential for evaluation of the tumor immune microenvironment and personalized treatment guidance. In particular, the potential impact of BNIP3 in osteosarcoma was further explored. Additionally forward-looking evidence for assessing the signature’s accuracy and applicability is required in the future.

## 5. Conclusions

This study elucidated the role and mechanism of BNIP3, PTGIS, and ZYX in OS progression and was well verified by the experimental results, enabling reliable prognostic means and treatment strategies to be proposed for OS patients.

This study established a signature (DGPS) composed of three genes for predicting osteosarcoma prognosis from a defense response perspective for the first time. DGPS had excellent performance in predicting one-, three-, and five-year survival rates and metastasis of osteosarcoma. It was also a strong predictor of survival in different clinical subgroups of osteosarcoma patients. The risk model DGPS we constructed taps into the relationship between osteosarcoma and the immune microenvironment; high-risk status classified by DGPS was associated with a reduction in immune infiltration. DGPS was also found to be instructive in the individualization of drug therapy for patients with osteosarcoma. BNIP3 was found to play an essential role in osteosarcoma metastasis and progression and was verified by apoptosis, proliferation, and migration experiments. Our findings have guiding significance in the clinical treatment and future research of osteosarcoma.

## Data Availability

These data were derived from the following resources available in the public domain: TARGET database (https://xena.ucsc.edu/, accessed on 6 April 2023), GEO database (https://www.ncbi.nlm.nih.gov/geo/, accessed on 6 April 2023).
